# Adverse drug events identified by triggers at a teaching hospital in Brazil

**DOI:** 10.1186/2050-6511-15-71

**Published:** 2014-12-13

**Authors:** Fabíola Giordani, Suely Rozenfeld, Mônica Martins

**Affiliations:** Department of Epidemiology and Biostatistics, Institute of Community Health, Fluminense Federal University, Marquês de Paraná Street 303, Annex HUAP 3rd floor, Niterói, RJ 24033-900 Brazil; Sérgio Arouca National School of Public Health, Oswaldo Cruz Foundation, Rio de Janeiro, Brazil

## Abstract

**Background:**

Adverse drug events (ADEs) are one of the most frequent causes of patient harm resulting from medical interventions, especially among inpatients. This study aimed to evaluate the incidence of ADEs and characterise them in terms of degree of harm, medication implicated and patient symptoms, at a Brazilian university hospital.

**Methods:**

This is a retrospective study of chart review. The method, developed by the Institute for Healthcare Improvement, uses triggers to identify possible ADEs. The study population comprised adult inpatients at least 15 years old. Obstetric patients and those hospitalised for less than 48 hours were excluded. Time spent in the intensive care unit was not considered for the purposes of this study. Patients were selected on the basis of simple random sampling of records of patients discharged from January to July 2008. The records selected were reviewed by a multidisciplinary team. The indicators of ADE incidence were patients with ADEs and ADE rate per 100 patients. Patients with and without ADE were compared in the bivariate analysis. To identify the drugs classes most often associated with events, the number of prescriptions of each class of drug was related to the number of events assigned to it.

**Results:**

The 240 inpatients studied were of mean age 50.8 (SD = 20.0) years, and mostly male (63.8%). A total of 44 ADEs were identified in 35 patient records, with 14.6% of patients presenting ADE and a rate of 18.3% ADEs per 100 patients. The most frequent were skin rash and nausea and vomiting, but severe ADEs were also identified. In the bivariate analysis long hospital stay and use of 10 or more drugs were associated with the occurrence of ADEs (p-value < 0.01). The drug classes associated with the highest number of events were anti-infective.

**Conclusion:**

About 1/6 of the hospitalized patients in a teaching hospital showed adverse events what is, by itself, cause for concern. Increased number of prescribed drugs and greater period of hospitalization appear to favour the occurrence of these events. In the future studies with higher number of patients may offer evidences of the association.

## Background

Adverse drug events (ADEs) are among the most frequent adverse events affecting hospital inpatients [1–3]. Percentages of hospital inpatients suffering ADEs range from 1.6% to 41.4% and the rate, from 1.7 to 51.8 events/100 admissions. A considerable proportion of such events are avoidable [4].

Despite a lack of consensus, important risk factors reported for ADEs include polypharmacy, female sex, administration of drugs with narrow therapeutic range, renal elimination of drug, age >65 years, and administration of anticoagulants or diuretics [5]. Other risk factors reported are acute diseases or metabolic disturbances, as well as use of drugs with low therapeutic indices and hepatic enzyme inhibitors or inducers [6].

Mechanisms for evaluating and monitoring the safety of drugs in clinical use are essential in order to prevent or reduce harm to patients [7]. There are various methods and techniques for identifying ADEs during hospital stay, including voluntary notification of cases, retrospective or prospective patient record review, and analysis of administrative data. An approach to identifying, quantifying and monitoring ADEs is to use triggers. These correspond to signs found during patient record review that may relate to adverse events [8, 9].

In Brazil this approach has been used in a few studies to identify events in general hospitals [10] and special units [11, 12].

Accordingly, the trigger method was used in this study to evaluate the incidence of ADE and characterise them in terms of degree of harm, medication implicated and patient symptoms, at a Brazilian university hospital.

## Methods

### Study design and population

This is a retrospective study of chart review at a public teaching hospital in the west of Paraná State in southern Brazil. The 173-bed hospital offers care to acute patients in various specialities.

Ethical approval for the study was given by the Ethical Committee of the State University of West Paraná (039/2009-CEP).

The study population comprised adult patients at least 15 years old. Obstetric patients and those hospitalised for less than 48 hours were excluded. Time spent in the intensive care unit was not considered for the purposes of this study. Patients were selected on the basis of simple random sampling of records of patients discharged from January to July 2008 (n = 1302). The parameters for calculating sample size were an estimated 15% of patients with ADEs, 95% confidence level and 10% desired absolute precision. Sample size calculation was 242.

Review of patient charts was performed with a tool developed by the Institute of Health Care Improvement (IHI) which consists of a set of triggers used to identify possible ADEs. The method is useful to measure the overall level of harm from medications in a health care organization. The trigger tool provides instructions for conducting a retrospective review of patient records. The records selected were reviewed to identify the presence of at least one of the 19 triggers proposed by the IHI [13].

### Definition of ADE

ADE was defined as any injury occurring during the patient’s drug therapy and resulting either from appropriate care, or from unsuitable or suboptimal care. The definition encompasses adverse drug reactions and medication errors [14].

### Data collection

The patient record review and evaluation were performed after training with the data collection instruments and instruction manuals. The reviewers discussed the methods and procedures intensively. The definitions of each trigger and possible associated ADEs were standardised. A pre-test of patient records not included in the sample helped standardise the data collection procedures. In addition, the reviewers were instructed to record any ADE identified during hospitalisation, even if the event was not associated with any trigger or was present on patient admission.

Evaluation of the adverse events was conducted in three stages:

In the first stage, the following information was extracted from the patient records: social and demographic data; drug prescriptions; and characteristics of the clinical and hospitalisation histories. The patient records were reviewed in the following order: laboratory results, drug prescriptions, and doctors’ and nurses’ clinical progress notes. Two reviewers (one pharmacy student and one medical student), working independently, evaluated each patient record. Divergences were resolved by a pharmacist with a background in public health.

The second stage was performed by a nurse and a pharmacist and comprised in-depth review of the records containing triggers. Those where at least one of these reviewers identified an ADE were selected for evaluation in the next stage.

In the third stage, we decided whether ADEs had occurred. The possible ADE was evaluated by the participants of the preceding stages, plus a clinician, in a face-to-face meeting.

Possible associations between events and suspect drugs were examined according to the drugs’ properties [15, 16], the patient’s clinical condition and the time until the occurrence of the event. The Naranjo algorithm [17] was applied to determine the strength of the causal relationship with the drugs used by the patient and implicated in the occurrence of each ADE. The total scores allowed us to classify the ADEs as doubtful (< 1), possible (1–4), probable (5–8) or definite (≥ 9).

The reviewers classified ADEs by degree of harm into five categories (E-I) [13]. The events were described using WHO Adverse Reaction Terminology (WHO-ART) [18].

Further information on the method and techniques employed can be found in a previous publication [19].

### Study variables and statistical analysis

The outcome was the ADEs occurring during the hospital stay. The variables evaluated were: age in years; sex (female; male); type of admission (acute/emergency; elective); type of treatment (surgical; clinical); type of discharge (medical discharge; transfer; death); treatment in intensive care unit (yes; no); length of stay in days, cut-off point by mean = 10.0 (SD = 12.2) days (2–10 days; 11 or more days); hospitalisation cost by tercile (low; middle; high); Charlson index (0; 1–2; and 3–9); number of drugs used (1–9; 10 or more); and medical diagnoses.

Drugs were coded according to the first and second levels of the Anatomical Therapeutic Chemical (ATC) classification [20]. The probability of association of each drug class with ADEs was calculated by dividing the number of times the class was positively related to an event by the number of prescriptions of each class, and multiplying by 100.

Co-morbidities – including the primary and secondary diagnosis from the cover sheet and coded according to the 10th International Classification of Diseases (ICD-10) [21], plus further co-morbidities identified from the progress sheets – were examined using the Charlson index [22]. The index evaluates patient severity by the presence of the following clinical conditions: AIDS, cerebrovascular disease, congestive heart failure, connective tissue disease, dementia, myocardial infarction, peripheral vascular disease, peptic ulcer disease, chronic obstructive pulmonary disease, hemiplegia, cancer, diabetes mellitus (with or without chronic complications); and liver and/or kidney disease.

The indicators of ADE frequency were: incidence of patients with ADEs (number of patients with at least one new ADE/number of patients) and ADE rate per 100 patients (number of new ADEs/number of patients) with their respective confidence intervals (95% CI). Patients with and without ADE were compared in the bivariate analysis. To identify the drugs classes most often associated with events, the number of prescriptions of each class of drug was related to the number of events assigned to it. To perform this the number of prescriptions of each drug class associated to an ADE was divided by the number of prescriptions of the drug class.

In the descriptive statistical analysis, the continuous variables were expressed by mean and standard deviation, and the categorical variables, by percentages. The continuous variables were subjected to the Kolmogorov-Smirnov test to examine the assumption of normal distribution. To test the differences between patients with and without ADEs, bivariate analysis was performed considering the independent variables. Where appropriate, the T-Student, Chi-square and Fisher’s Exact tests were performed to compare sub-groups.

Data were processed using EpiData 3.0 and Microsoft Office Access 2003, and analysed using the statistical packages SPSS 15.0 for Windows ® (SPSS Inc., Chicago, IL, U.S.A.) and R version 2.11.1.

## Results

### Characteristics of participants

The sample calculated comprised 242 patients. Of these there were nine losses, seven of which were considered replaceable (three patients received outpatient treatment and were not hospitalised; two remained in the ICU throughout their hospitalisation; one was an obstetric patient; and one spent less than 48 hours hospitalised). The other two losses were deemed irreplaceable (patient records not located). These hospitalisations comprised 238 patients, but two were re-admitted and these were considered independent events. Thus the final sample comprised 240 records of patients admitted between January and July 2008 (corresponding to 2408 patient-days), whose data were examined.

The primary diagnoses most often found included “injury, poisoning and certain other consequences of external causes”, followed by “diseases of the digestive system” and “diseases of the circulatory system”.

The patients’ mean age was 50.8 years, very close to the median (50 years), and they were predominantly male (63.8%). In the study sample, most of the patients (79.6%) were acute or emergency admissions; approximately 62.0% received surgical treatment and 5.4% died. Seventy-five percent of the patients were hospitalised for less than 10 days and mean hospital stay was 10.0 (SD = 12.2) days. In the sample, 60.0% of the patients displayed no co-morbidities contributing to the Charlson index and 51.7% used, on average, 10 or more different drugs during their hospitalisation. In the bivariate analysis only long hospital stay and use of 10 or more drugs were associated with the occurrence of ADEs (p-value < 0.01) (Table 1).

**Table 1:** **In-patient characteristics by occurrence of Adverse Drug Events (ADEs) at a Brazilian hospital**

Variable	Without ADE	With ADE	Total	P-value ^a^
	N (%)	N (%)	N (%)	
**Total**	205	35	240	-
**Age in years**	50.6 (19.8)	51.6 (21.4)	50.8 (20.0)	0.788
Mean (standard deviation)				
**Sex**				
Female	71 (81.6)	16 (18.4)	87 (36.2)	0.208
Male	134 (87.6)	19 (12.4)	153 (63.8)	
**Nature of the admission**				
Urgency/emergency	162 (84.8)	29 (15.2)	191 (79.6)	0.603
Elective	43 (87.8)	6 (12.2)	49 (20.4)	
**Type of treatment**				0.09
Surgical	131 (88.5)	17 (11.5)	148 (61.7)	
Clinical	74 (80.4)	18 (19.6)	92 (38.3)	
**Status of discharge**				
Discharged/transferred	195 (85.9)	32 (14.1)	227 (94.6)	0.290
Death	10 (76.9)	3 (23.1)	13 (5.4)	
**ICU**				
Yes	11 (84.6)	2 (15.4)	13 (15.4)	0.933
No	194 (85.5)	33 (14.5)	225 (94.6)	
**Length of stay** ^**b**^				
≤ 10 days	164 (91.1)	16 (8.9)	180 (75.0)	<0.001
> 10 days	41 (68.3)	19 (31.1)	60 (25.0)	
**Cost of hospitalisation** ^**c**^				
Low	73 (90.1)	8 (9.9)	81 (33.8)	0.331
Medium	66 (82.5)	14 (17.5)	80 (33.8)	
High	66 (83.5)	13 (16.5)	79 (32.9)	
**Charlson index**				
0	126 (87.5)	18 (12.5)	144 (60.0)	0.084
1-2	55 (87.3)	8 (12.7)	63 (26.3)	
3-9	24 (72.7)	9 (27.3)	33 (13.8)	
**Number of drugs**				
1-9	117 (94.4)	7 (5.6)	124 (48.3)	<0.001
10 or more	88 (75.9)	28 (24.1)	116 (51.7)	
**Total**	205	35	240	-

^a^Using T-Student for comparing mean ages, Chi-square and Fisher’s Exact tests for comparing categorical variables.

^b^Cut-off by mean (10.0).

^c^Categorised in tertiles.

### Incidence and rate of ADEs

A total of 44 ADEs were identified in 35 patient records, with 14.6% (95% CI 10.1-19.1) of patients presenting ADE and a rate of 18.3% (95% CI 13.4-23.2) ADEs per 100 patients. Of the 35 patients with ADEs, seven showed two or more events, with two of them showing three. Among the ADEs identified, two were not associated with the triggers (one was increased transaminases from use of phenytoin and the other was urinary retention from use of chlorpromazine). Two other events led to patient hospitalisation and did not enter into the analyses. In nine ADEs, identification was made possible by the presence of two or more triggers. One ADE, a skin rash, was identified by three different triggers: “antiallergics”, “skin rash” and “abrupt discontinuation of medication”.

### Characteristics of ADEs

Using the Naranjo algorithm, 23 (52.3%) of the ADEs were classified as “possible” and 20 (45.4%), as “probable”. In one case (2.3%), it was not possible to apply the algorithm.

As regards patient harm produced, 37 events (84.1%) were classified in category E, as involving temporary harm requiring intervention, and 4 (9.1%) in category F, as causing temporary harm and more extended hospital stay. Three (6.8%) events were classified as H, where the harm demands intervention to keep the patient alive; these were hypoglycaemia, cardiac tamponade and over-sedation.

Table 2 describes the events. The most frequent were skin rash (8 events), where ranitidine figured prominently as the attributed drug, and nausea and vomiting (8 events) associated mainly with the use of an opioid (nalbuphine) and anti-infectives. Severe events affecting other systems included prolonged hypoglycaemia, cardiac tamponade and bed fall.

**Table 2:** **Adverse drug events (ADEs) and imputed drugs**

Description ADE ^a^	Number of cases (n = 44)	Drugs
Rash	8	Ranitidine. Metronidazole. Cefazolin. Omeprazole. Morphine. Drug undetermined
Nausea and/or vomiting	8	Nalbuphine. Amphotericin B. Omeprazole. Mannitol. Moxifloxacin
Pruritus, rash, and dizziness	4	Tenoxicam. Nalbuphine. Cefalotin
Bleeding (haemoptysis, bleeding or melena)	4	Warfarin. Heparin. Omeprazole. Amitriptyline. Simvastatin. Heparin
		Acetylsalicylic acid. Heparin
Somnolence	2	Chlorpromazine. Phenytoin
Diarrhoea	2	Ampicillin + sulbactam. Lactulose
Tremor	2	Metoclopramide. Moxifloxacin
Cardiac tamponade	1	Heparin
Fall (from bed)	1	Captopril. Hydrochlorothiazide
Seizure	1	Methylprednisolone
Pseudomembranous colitis	1	Cefepime. Clarithromycin
Hypoglycaemia	1	Insulin
Excessive sedation	1	Midazolam
Other	8	Captopril. Furosemide. Nalbuphine, Morphine. Amphotericin B. Sulfamethoxazole + trimethoprim. Cefepime. Fluconazole. Pentamidine. Phenytoin. Chlorpromazine.

^a^The events were described using WHO Adverse Reaction Terminology (WHO-ART).

The drug classes associated with the highest number of events were those used for the nervous system (15), anti-infectives for systemic use (13) and drugs used to treat problems of the alimentary tract and metabolism (10) and blood and blood forming organs (7), as a result of their being the most prescribed. The drug class most strongly associated with ADEs was antiparasitic products (12.5%). The drug sub classes most prescribed were analgesics, antibacterial for systemic use, antithrombotic agents and drugs for acid related disorders (Table 3). The drugs most commonly involved were nalbuphine (5), heparin (4), ranitidine (4), captopril (2), phenytoin (2), chlorpromazine (2), morphine (2), moxifloxacin (2), amphotericin B (2) and omeprazole (2).

**Table 3:** **Drug classes related to an adverse drug event (ADE), by anatomical therapeutic chemical classification (ATC)**

ATC code ^a^	Number of prescriptions of the drug class	Number of prescriptions of the drug class associated with an ADE ^b^	Proportion (%)
**A - Alimentary tract and metabolism**	**478**	**10**	**2.09**
A02 - Drugs for acid related disorders	197	7	3.6
A03 - Drugs for functional gastrointestinal disorders	182	1	0.6
A06 – Laxatives	15	1	6.7
A10 - Drugs used in diabetes	59	1	1.7
**B - Blood and blood forming organs**	**354**	**7**	**2.0**
B01 - Antithrombotic agents	228	6	2.6
B05 - Plasma substitutes and perfusion solutions	102	1	1.0
**C - Cardiovascular system**	**277**	**5**	**1.8**
C03 – Diuretics	69	2	2.9
C09 - Agents acting on the renin-angiotensin system	79	2	2.5
C10 - Lipid modifying agents	28	1	3.6
**H - Systemic hormonal prep, excluding sex hormones**	**44**	**1**	**2.3**
H02 - Corticosteroids for systemic use	44	1	2.3
**J - General antiinfectives for systemic use**	**405**	**13**	**3.2**
J01 - Antibacterials for systemic use	387	10	2.6
J02 - Antimycotics for systemic use	7	3	42.9
**M - Musculo-skeletal system**	**177**	**1**	**0.6**
M01 - Antiinflammatory and antirheumatic products	168	1	0.6
**N - Nervous system**	**566**	**15**	**2.7**
N02 – Analgesics	419	9	1.7
N03 – Antiepileptics	40	2	5.0
N05 – Psycholeptics	82	3	6.1
N06 - Psychoanaleptics	11	1	9.1
**P - Antiparasitic products**	**8**	**1**	**12.5**
P01 – Antiprotozoals	2	1	50.0
**Unclassified drug**	**9**	**1**	**11.1**
**Total**	**2499**	**54**	**2.2**

^a^The first and second level of the Anatomical Therapeutic Chemical Classification (ATC) associated with at least one ADE.

^b^Number of ADE-drugs is greater (54) than number of ADEs (44), because one drug can be associated with several ADEs.

## Discussion

About on-sixth of inpatients were found to have experienced ADEs, at a mean rate of 18.3 ADEs per 100 patients. A meta-analysis of observational studies presented estimates of ADE according to the method of identification of events [23]. In two out of twenty five studies the events were identified by a similar set of triggers. One of them was conducted in the United States with six community hospitals and estimated a rate of 15.0 ADEs per 100 patients [24]. In the other study, researchers encountered in a Brazilian hospital a rate of 26.6 ADEs per 100 patients [10]. Although the event rate in the last study is higher than ours the proportion of people with ADE estimated by the authors [10] is similar, around 15%. Some characteristics of the study population and the hospital’s profile may explain the differences. The differences in rates may be also attributed to staff education or case mix that may occur even when comparing data from a single country.

The characteristics of ADEs we focused on were patient symptoms and degree of harm. Rashes, nausea, vomiting, pruritus and dizziness accounted for almost half the events. The profile of ADEs identified in our study is similar to that identified in a study conducted in a tertiary care hospital in Northern Brazil, where skin was found to be the most commonly affected organ system. The gastrointestinal system was also among the three most affected of them [25].

As regards degree of harm, most of the events resulted in temporary patient harm that required some intervention. Other studies also used the same source to classify ADEs, which is the National Coordinating Council for Medication Error Reporting and Prevention Index. They obtained similar proportions of events of lower degree of severity, 87% [26] and 79.9% [8]. Life-threatening events were much less common, as in other studies [24]. We also identified events that required intervention to keep the patient alive, such as hypoglycemia, cardiac tamponade and over-sedation, related, respectively, to the use of insulin, heparin and midazolam.

In our sample, certain classes of drugs are intensively prescribed and also strongly associated with ADEs (i.e., return higher ratios of “related ADEs” to “number of prescriptions”), they were analgesics, antibacterials for systemic use, antithrombotic agents and drugs for acid-related disorders. Ranitidine is a drug from this last subclass, as it is a histamine H2 receptor antagonists, and serves to illustrate the problem.

Ranitidine was implicated in cases of rash. It was the most frequent event identified in our study. It was prescribed for 70% of patients, a value compatible with those found in the literature for use in therapy to suppress gastric acid production [27, 28]. We observed that, in most patients, ranitidine was being used as prophylactic medication rather than to treat gastrointestinal diseases. Stress ulcer prophylaxis has become an increasingly common practice for clinical patients, although there is little or no clinical evidence to support it [27] and it can be considered unnecessary in 73% of cases [28]. According to figures from this hospital, more than 50% of the ranitidine dispensed is for intravenous administration, which exposes the patients to unnecessary risk, because the injection route can cause local burning, itching, skin rash and vasculitis [15].

The occurrence of ADEs is associated with the number of drugs used [5, 25, 29–31]. Our data corroborate that association: the likelihood of an ADE occurring was higher in users of 10 or more drugs than in those who used fewer drugs during their hospital stay (p value <0.001), although the analysis was not adjusted for confounders. When patients are seriously ill, it is often difficult to evaluate the degree of harm to be attributed to the number of prescribed drugs or to drug classes. In our study population, one patient suffered a bed fall during the hospital stay. Besides the fact that he was exposed to drugs likely to cause falls (captopril, hydrochlorothiazide), evaluation of the harm was jeopardised by the complexity of the patient’s condition (serious traffic accident casualty).

Patients with longer hospital stays are exposed to greater likelihood of ADE than those hospitalised for up to nine days (p value <0.001). This finding is consistent with those of several previous studies [5]. Hence it is important draw attention to long-stay inpatients, because in addition to being prone to ADEs, the tendency is towards multiple events and more pronounced patient harm.

As longer hospital stays and use of more drugs are regarded as factors associated with ADEs, our findings may help physicians to identify at-risk patients and monitor them carefully. Moreover, to prevent ADES during hospital stay, the risk factors can be categorised according to opportunities for intervention, such as decreasing the number of drugs prescribed or adjusting the dose [32].

This study has limitations in terms of the internal validity. The results of specific events should be analysed with caution because the sample size was calculated considering the estimated global incidence of ADEs. Thus, the frequency of specific events may reflect random variations and may be skewed.

Although the study is not large, we did succeed in observing values of the estimates that differed with statistical significance between patients with or without ADEs for the variables length of stay and number of medications in the bivariate analysis. However studies with larger samples should test the hypothesis of association.

The study was conducted at only one hospital, which makes external validity troublesome and places limitations on how far the results can be generalised. Nonetheless, they may be applied to other tertiary hospitals in medical schools in Brazil and in other countries.

We endeavoured to increase internal validity by using double data collection and assembling a team of researchers and health professionals to evaluate the cases. In order to improve objectivity, the method was applied at all stages by two independent reviewers, using a specific manual containing definitions and detailed descriptions of the procedures. Nonetheless, some subjectivity in identifying and interpreting triggers and events cannot be ruled out, as demonstrated in other retrospective patient record review studies [33]. The double-blind evaluation performed here was not intended to assess inter-observer reliability, but rather to foster information completeness and improve validity of the information. The double evaluation process with two physicians of patient records to assess ADE is not more reliable than a record review process with one physician [34].

Poor quality of information in hospital records is a common problem in chart review-based research [35], but the choice of a teaching hospital possibly reduces information bias. There were concerns about data completeness, however. Conspicuous gaps in the recorded information included race/ethnicity and occupation, which were missing from about 20% of patient records.

During application of the method, two events unrelated to the triggers were identified, one of them a case of increased blood transaminase levels resulting from use of phenytoin. Studies have shown that laboratory test results for transaminases levels can be effective triggers for detecting ADEs. Aspartate aminotransferase (AST) screening returns a positive predictive value between 0.01 and 0.23, and alanine aminotransferase (ALT), a value from 0 to 0.31 [36]. The fact that, in our study, some ADEs were not found by triggers reinforces the idea that no single method captures every event that occurs in hospitals. This challenges managers and policy makers to test and harmonise different methods to address adverse events. The use of triggers to identify ADEs, when integrated with event monitoring, stimulated or unstimulated spontaneous reporting and other techniques, can be an important strategy for indicating possible shortcomings in the process of using medications for hospitalised patients.

According to Rozich [8] the trigger enables organisations to monitor longitudinally how ADE rates change in response to strategies designed to improve clinical safety [8]. However, this strategy is nowadays mainly theoretical, since there are several obstacles to be overcome, among them: scant evidence of impact of ongoing strategies; the existence of different subcategories of drug adverse events requiring different improvement interventions; the fact that many common adverse events require more complex detection strategies [37, 38].

The concept of ADE we used included drug adverse reactions (ADR) and errors. It does not conflict with the concept of ADR that sustains the algorithm used to determine the strength of the causal relationship with the drugs in our study and others [39–41]. Steady conceptual advances in the field of pharmacoepidemiology are permitting studies focussing primarily on occurrences of patient harm, regardless of whether or not such harm is associated with errors in therapeutic indication or drug administration. Besides, examining carefully the patient charts we did not identified any case of error like non adherence and subtherapeutic doses.

In 2013 Brazil’s Ministry of Health launched a national patient safety programme [42]. It hinges on engaging patients in health care, including the subject of ‘patient safety’ in undergraduate and postgraduate education, and increasing research. On this latter item, the programme specifies measuring harm, understanding the causes, identifying the solutions, assessing the impact and applying the evidence to assure safer patient care. Given that framework, we believe the results of this study can help to call attention to the need of develop researchs aiming to estimate and characterize ADE and medication involved as well as the role of number of drugs prescribed for inpatients.

## Conclusion

This study draws attention to the problem of ADE in hospitalized patients and offers a methodological alternative for future research in Brazil as well as other underdeveloped countries. Our results suggests that trigger tool may be useful to identify ADE in hospitals.

About 1/6 of the hospitalized patients in a teaching hospital showed adverse events what is, by itself, cause for concern. Although the events were classified as less serious in over 80% of cases, we also identified additional and more severe events that required intervention to keep the patient alive.

Analgesics, antibacterials for systemic use, antithrombotic agents, drugs for acid-related disorders should be used sparingly, because they are very often prescribed and associated with the highest numbers of events. These results should be examined with caution, as the number of ADE is small (44/240 patients). Increased number of prescribed drugs and greater period of hospitalization appear to favour the occurrence of these events but additional research should test this hypothesis.
